# Patches of Bare Ground as a Staple Commodity for Declining Ground-Foraging Insectivorous Farmland Birds

**DOI:** 10.1371/journal.pone.0013115

**Published:** 2010-10-06

**Authors:** Michael Schaub, Nicolas Martinez, Aline Tagmann-Ioset, Nadja Weisshaupt, Melanie L. Maurer, Thomas S. Reichlin, Fitsum Abadi, Niklaus Zbinden, Lukas Jenni, Raphaël Arlettaz

**Affiliations:** 1 Division of Conservation Biology, Institute of Ecology and Evolution, University of Bern, Bern, Switzerland; 2 Swiss Ornithological Institute, Sempach, Switzerland; University of Hull, United Kingdom

## Abstract

Conceived to combat widescale biodiversity erosion in farmland, agri-environment schemes have largely failed to deliver their promises despite massive financial support. While several common species have shown to react positively to existing measures, rare species have continued to decline in most European countries. Of particular concern is the status of insectivorous farmland birds that forage on the ground. We modelled the foraging habitat preferences of four declining insectivorous bird species (hoopoe, wryneck, woodlark, common redstart) inhabiting fruit tree plantations, orchards and vineyards. All species preferred foraging in habitat mosaics consisting of patches of grass and bare ground, with an optimal, species-specific bare ground coverage of 30–70% at the foraging patch scale. In the study areas, birds thrived in intensively cultivated farmland where such ground vegetation mosaics existed. Not promoted by conventional agri-environment schemes until now, patches of bare ground should be implemented throughout grassland in order to prevent further decline of insectivorous farmland birds.

## Introduction

Farming practices have changed radically since World War II, provoking an unprecedented crisis for farmland biodiversity [1]. First, the total area devoted to agricultural production has increased through the conversion of pristine habitats into grassland or arable land [2]. Second, natural elements constituting the matrix of traditional agricultural landscapes have vanished: wetlands have been drained, streams channelized or contained in underground pipes, while patches of forest, hedges and grassy field margins have been eradicated [3]. Third, fertilizers have substantially increased the yields per unit of land and time: the resulting sward thickening has changed micro-climatic conditions within grassland, thereby lowering invertebrate abundance and reducing accessibility for many organisms [4], [5]. Fourth, the systematic application of pesticides and herbicides has eliminated plant and animal species not perceived as directly useful for agricultural production [6]. Overall, agricultural development has dramatically increased the human share of net primary productivity (NPP) at the biosphere scale (currently about 50% of continental NPP [7]). This diversion of NPP for the sake of humans has benefited our rapidly growing population by substantially increasing food supply; however, it has also caused large-scale biodiversity erosion.

New policies aimed at restoring farmland biodiversity were thus launched in most developing countries. The basic idea was, first, to restore natural elements within the agricultural matrix; second, to set aside part of the land used so as to make a substantial proportion of NPP again available for living forms other than humans; and, third, to extensify agricultural practices through a reduction in agrochemical and fertilizer application in order to better preserve water, soil and air. Implemented in several countries, such agri-environmental measures have so far only moderately supported biodiversity [8], [9], [10], [11], [12], [13], [14]: although common species at the lower trophic levels have benefited on a local level, rare species, which are often situated higher up in the food chain, have seen their numbers stagnate or even further decline.

Farmland birds have suffered massive population declines over the past decades [15], especially in the industrialized countries, and this trend continues [16], now even affecting remote mountainous areas [17]. At the time they were launched, agri-environment schemes carried much hope for an improvement in farmland birds' status, although they were mostly designed for the wider countryside, i.e. thought as landscape-focused schemes. Yet, more than one decade after the widespread implementation of agri-environment policies, few examples of population recoveries have been documented. Most studies have detected limited or moderate effects [14], [18], [19], which led to intense public debates about the relevance of agri-environment schemes for promoting biodiversity in general [8]. Ground-foraging insectivorous birds have been especially affected by agricultural changes [20] and they typically do not respond positively to existing agri-environment schemes [19]. The reason for this may be manyfold. First, food biomass supplied by current agri-environment schemes in breeding areas may be insufficient to compensate for losses due to the intensification of farming practices [4], i.e. a suitable food supply has not been restored for these birds. Second, due to changes in vegetation structure, food resources may be present in sufficient quantity on breeding grounds but remain largely inaccessible, while birds may face an increased predation risk [21], [22]. Other reasons than resources availability on breeding grounds may also play a role, e.g. deterioration of environmental conditions on wintering grounds, rendering these agri-environment schemes useless. All these factors are of course not mutually exclusive. In this study, we addressed the first two aspects. We assessed fine-grained habitat selection in four declining species of European ground-feeding insectivorous birds in various types of high intensity farmland. At our study sites, these birds – that historically had their population strongholds in traditional, low intensity farmland – still survive in high intensity agricultural matrices (fruit tree plantations, orchards and vineyards). By recognizing convergences in basic ecological requirements between species and across farmland types this study aimed to identify one possible main reason why current agri-environment schemes fail to promote these terrestrially feeding insectivorous birds, and to recommend new management measures in order to improve the schemes.

## Materials and Methods

We studied foraging patch selection of adult hoopoes (*Upupa epops*), wrynecks (*Jynx torquilla*), woodlarks (*Lullula arborea*) and common redstarts (*Phoenicurus phoenicurus*) providing food to chicks. The studies were conducted in Southwestern Switzerland (Valais near Sion, 46° 41′N, 7° 22′E; hoopoes, wryneck, woodlark) and in Northern Switzerland (Basel, 47° 33′N, 7° 35′E; common redstart). The dominant habitats in the study areas were intensively farmed fruit tree plantations (hoopoe, wryneck), intensively cultivated vineyards (woodlark) and high-stem orchards in dense, mostly intensively managed grassland (common redstart).

Because detectability of ground-foraging birds is generally low and declines with increasing vegetation cover, we relied on radiotracking for three study species (hoopoe, wryneck, woodlark). This ensured unbiased results regarding the relationship between ground vegetation structure and foraging behaviour. Radio-tracked birds were equipped with light radiotags (BD-2-P with activity sensor, 0.9–1.4 g, Holohil Systems Ltd., Canada) fitted using a leg-loop harness [23]. We used the homing-in technique to approach a focal bird as soon as we got a pulse-rate alternating signal indicating foraging [24]. We eventually aimed at recording its precise foraging location visually, while avoiding to disturb its activities during the approach. To avoid temporal autocorrelation of location data, we only considered consecutive foraging locations that were recorded at least 5 minutes apart, unless the bird had moved to another foraging site in between. Capture and radiotracking were performed under authorizations of the Swiss Ministry for the Environment and the Valais Cantonal Office for Fisheries and Wildlife, in accordance with the Federal Law of 20 June 1986 on Hunting and the Protection of Wild Mammals and Birds. For common redstarts, foraging locations were obtained from visual observation as the species' sit-and-wait foraging tactic renders them more conspicuous. Therefore, the identification of feeding locations was unrelated to the ground vegetation structure.

Individual home ranges were delineated as the minimum convex polygons encompassing all foraging locations of a given individual. Within each individual home range we then randomly selected a number of points using ArcView (ArcView GIS 3.3, ESRI). The random selection was performed in such a way that these points did not fall within a circle of 10 m radius around the foraging locations. The number of randomly selected points closely matched the number of observed foraging locations in each individual home range, but the number of recorded points differed between study species (Table S1). At each point (foraging location or random point), and within a circle of 1 m radius (5 m in woodlarks), we estimated the proportion of bare ground visible when looking vertically down onto the ground, as well as the average height of the ground vegetation (except for the common redstart, where vegetation height was not measured). We also recorded the habitat type (fruit tree plantation, orchard, vineyard, meadow, pasture, wood, and cropland) to which the points belonged to. In total we identified 1’471 foraging and 1’417 random locations for 33 individuals (13 hoopoes, 8 wrynecks, 7 woodlarks, 5 common redstarts, Table S1).

We analysed the data separately for each species, applying a hierarchical logistic regression model (with random intercept and slope parameters) implemented in a Bayesian framework using Markov chain Monte Carlo simulation (Appendix S1). The response variable was Boolean, with a 0 value for random locations and 1 for foraging locations. The reliance on a hierarchical design [25] circumvented the problem of traditional habitat selection analyses that requires running separate analyses for each individual in order to obtain data independence, while it enabled the recognition of any species-specific pattern across individuals. Furthermore, this approach also made it possible to fit a functional response for each individual as well as deriving a marginal response at the population level.

For each dominant habitat category for hoopoe (fruit tree plantation, grassland, all remaining habitat types together) and wryneck (fruit tree plantation, all remaining habitat types together) we first ran the basic model including effects of bare ground and its square, as well as vegetation height and its square. This enabled us to evaluate whether the relationships between bird occurrence and vegetation structure were consistent among broad habitat categories. We found that this was the case (Figure S1), and thus did not consider habitat categories in subsequent analyses. For woodlarks and common redstarts the vast majority of locations occurred in one habitat type only for each species (vineyard and orchard, respectively).

Second, we fitted different models that included different combinations of effects of bare ground and its square, as well as vegetation height and its square. The models were then ranked according to the deviance information criterion (DIC, [26]). Squared effects were included because of a likely trade-off between food abundance and accessibility on the one hand, and vegetation density and height on the other, which would result in curvilinear relationships peaking at intermediate values of predictor variables.

Based on the best models we calculated predictive distributions to evaluate goodness-of-fit. We compared observed values with predicted values using χ^2^-diagnostics and report Bayesian *P*-values. If the fit of the model was good, Bayesian *P*-values around 0.5 were expected [27].

## Results

**Figure 1:**
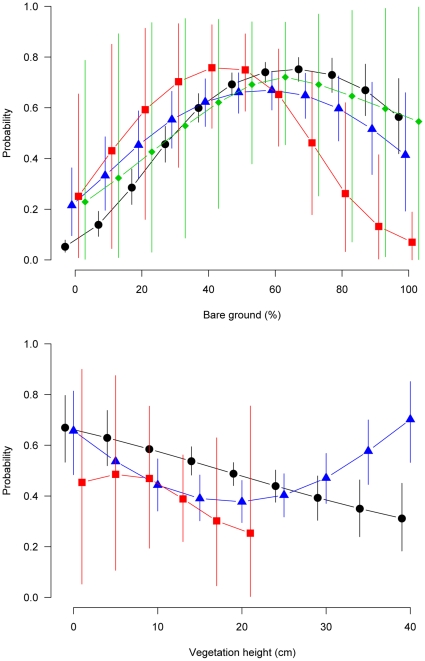
Marginal selection probability of foraging locations in relation to amount of bare ground and vegetation height for four farmland bird species. Predictions are revealed from the best models (see the supporting information) and refer to hoopoes (black dots), wrynecks (blue triangles), woodlarks (red squares) and common redstarts (green diamonds). Note that selection probabilities below 0.5 indicate avoidance, selection probabilities above 0.5 indicate preference. Points are posterior means, vertical lines show the limits of the 80% credible intervals.

The habitat selection analysis showed that both variables characterizing ground vegetation structure, i.e. proportion of bare patches on the ground and vegetation height, were important in determining the presence of a foraging species (Table 1, see also Figure S2 for individual effects and Table S2 for parameter estimates). In all species, there existed a quadratic relationship between occurrence of foraging birds and amount of bare ground, with an optimum of 30–70% bare ground at the foraging patch scale (∼3 m^2^, Figure 1). Within species, the shape of the functional response curve was similar in all individuals and it was consistent across habitat types (Figure S1), thereby identifying bare patches as a staple commodity for these individuals and species. Vegetation height was examined for three species, and it was clearly found to be of lesser importance than the amount of bare ground, as evidenced by the predictions (Figure 1). Furthermore, species reacted differentially to varying vegetation height. Woodlarks and hoopoes favoured places with shorter swards while wrynecks did not show preference for any particular sward height.

**Table 1:** Model selection results for the effect of the proportion of bare ground (b), its square (b^2^), the vegetation height (h) and its square (h^2^) on the foraging selection probability for the four bird species.

	Hoopoe			Wryneck			Woodlark			Common redstart		
Model	Deviance	pD	ΔDIC	Deviance	pD	ΔDIC	Deviance	pD	ΔDIC	Deviance	pD	ΔDIC
b+b^2^+h+h^2^	998.64	34.53	1.36	**351.32**	**18.39**	**0.00**	**935.82**	**32.26**	**0.00**	-	-	-
b+h+h^2^	1123.13	30.56	121.88	359.73	18.85	8.86	1056.36	26.96	115.24	-	-	-
h+h^2^	1327.08	21.25	316.51	386.66	10.54	27.48	1268.55	21.17	321.64	-	-	-
b+b^2^+h	**1000.02**	**31.78**	**0.00**	357.78	16.97	5.04	951.48	26.69	10.09	-	-	-
b+b^2^	1071.58	20.09	59.87	363.92	11.09	5.29	1052.83	18.74	103.50	**544.34**	**11.39**	**0.00**
b+h	1145.17	26.89	140.26	363.16	15.87	9.31	1093.14	20.45	145.51	-	-	-
b	1231.21	13.99	213.39	369.74	9.24	9.26	1185.16	12.94	230.03	551.46	9.97	5.69
h	1356.06	13.53	337.78	391.20	8.54	30.03	1315.51	13.11	360.54	-	-	-
intercept	1451.73	2.21	422.13	398.49	2.16	30.93	1399.41	2.01	433.33	750.66	2.06	196.99

Given are the deviance, the model complexity (pD) and the difference of the deviance information criterion between the best and the current model (ΔDIC). The best models are bold printed. The goodness-of-fit tests of the best models were acceptable in all species (Bayesian *P*-values, hoopoe: 0.54, wryneck: 0.19, woodlark: 0.24, common redstart: 0.32).

## Discussion

The common preference seen in all four bird species for bare ground across the different types of farmland habitats suggests that food availability is of paramount importance for habitat selection [28], [29]. Thus, food availability (i.e. prey abundance modified by its accessibility) is *per se* a crucial, limiting resource for ground-feeding insectivorous birds [30]. On one hand, ground vegetation provides the invertebrate food biomass for the birds, which can detect and pick up prey items from the bare patches on which they can easily manoeuvre in the absence of obstacles [31], [32], [33]. The question then arises as to how abundant the prey supply must be in the ground vegetation and how patches of bare ground must be distributed within the agricultural matrix to offer suitable conditions for these birds. The fact that we worked in high intensity farmland (dwarf fruit plantations and vineyards which are regularly treated with pesticides) suggests that prey abundance may be less important than previously thought. An experimental examination on caged common redstarts also showed that hunting individuals preferred sparsely vegetated patches with low food supply over densely vegetated patches with high food supply [34]. We think, however, that the best compromise is obtained when ground vegetation harbours abundant populations of invertebrates, which is rarely the case when the grass is either maintained short or is lacking completely over the whole surface [35]. This compromise would be best achieved by a spatially fine-grained mosaic of patches of grass and bare ground within the agricultural matrix.

A further advantage of foraging in sparse and short vegetation is a reduced risk of predation [22]. The greater the visibility a prey has, i.e. the fewer obstructions (e.g. long or dense vegetation; [36]), the faster the prey is likely to detect and respond to predators. Since the risk of predation can alter foraging behaviour and thereby discourage individuals from foraging on patches which otherwise offer the largest amount of prey, fitness may be affected, even though the birds do not in fact experience frequent predation [37]. Increased prey accessibility and avoidance of predation risk are the two reasons for which ground foraging insectivorous birds appear to prefer foraging in sparse vegetation, despite higher food abundance in dense vegetation. These two reasons are, of course, not mutually exclusive.

Interestingly, current management of most fruit tree plantations and some modern vineyards at the Valais study sites seems to offer the appropriate mosaic at the foraging patch scale. Depending on culture type, a varying proportion of the grass layer is destroyed by herbicide application or mechanical removal along tree or vine lines to avoid competition for water between ground vegetation and fruit plants. In several instances, the current proportions of bare ground and grass at the site scale seem to offer suitable conditions for these rare birds which have stable populations in most fruit tree plantations and in those vineyards which are ground-vegetated. Current management should therefore preferably continue in fruit tree plantations, while vegetated vineyards, which are progressively replacing conventional mineral vineyards in Valais, should be further promoted.

Mermod et al. [38] and Coudrain et al. [39] both provided evidence that bare ground is important also at the territory scale, but the optimal proportion (∼30–50%) was less than at the foraging patch scale. This suggests that a suitable breeding ground does not necessarily need to have a fine-grained grassy-bare mosaic throughout, and that a few bare patches may already offer attractive conditions. Yet, for many farmland habitats characterised by a dense and continuous grass cover, further studies are necessary to evaluate the optimal arrangement of vegetated and bare patches at the breeding ground scale. Ideally such studies should not only focus on habitat use, but also on fitness correlates.

We conclude that ground-feeding insectivorous farmland birds prefer to forage on patches of bare ground within grassy habitats. The dense sward that characterizes both modern, fertilized grassland and most grassy ecological compensation areas ([10], low-intensity and extensive meadows, set-aside land, wildflower areas, etc.) in restored agricultural matrices does not match the requirements of these bird species. This calls for a change of management to restore appropriate cultivated landscapes. More open vegetation can be achieved despite general nitrogen and carbon enrichment on the soil surface [40], [41]. First, by extensifying grassland management (less fertilization and irrigation) patches of bare ground can be reinstated within cultivated habitats. Second, mechanical or chemical removal of the ground vegetation cover could be conducted in grassy habitats where extensification is difficult to achieve (e.g. set-aside and wildflower areas). Pros and cons of herbicide application should be carefully evaluated, taking into account not only implications for the environment (air, soil, water) but also for biodiversity. By integrating these measures, future agri-environment schemes could benefit threatened species of insectivorous farmland birds as well as many other organisms that profit from habitat heterogeneity at the site scale [3].

## Supporting Information

Appendix S1 (0.04 MB DOC)

Figure S1 Selection probability of habitat use in relation to amount of bare ground and vegetation height for hoopoe and wryneck in different habitat categories as revealed by the most complex model. The grey lines show the individual effects, the black and blue line shows the population (marginal) average with 80% credible intervals. Note that selection probabilities below 0.5 indicate avoidance, selection probabilities above 0.5 indicate preference.
(2.92 MB TIF)

Figure S2 Selection probability of habitat use in relation to the amount of bare ground and vegetation height for four farmland species as revealed by the best models (Table 1). The grey lines show the individual effects, the coloured lines show the population (marginal) average with 80% credible intervals. Note that selection probabilities below 0.5 indicate avoidance, selection probabilities above 0.5 indicate preference.
(2.33 MB TIF)

Table S1 Sample sizes, locations and the use of radio-tags for the four studies: number of individuals, total number of observations and random points, and mean number of observations and random points per individual.
(0.04 MB DOC)

Table S2 Estimates of the mean model parameters and of their variability among individuals from the most complex model (b+b2+h+h2) for each species. Values in parentheses show the limits of the 80% credible intervals for each estimate.
(0.05 MB DOC)
