# Supplementary material for: Patches of Bare Ground as a Staple Commodity for Declining Ground-Foraging Insectivorous Farmland Birds
Source: PLoS One. 2010 Oct 6;5(10):e13115. doi: 10.1371/journal.pone.0013115 (PMC2950849; doi:10.1371/journal.pone.0013115)
Supplement: Appendix S1 — (0.04 MB DOC) [file pone.0013115.s001.doc]

### Appendix S1: Statistical model

We used a hierarchical logistic regression model implemented in a Bayesian framework to analyse the binary response variable (y). For each individual j (j = 1…J) and each observation i (i = 1…I), the dependent variable y_i,j_ follows a Bernoulli distribution:

$$y_{i,j}\sim\text{Bern}\left(\mu_{i,j}\right)$$

The expected value μ_i,j_ is modelled by variables describing the structure of the ground vegetation using the logit link function:

$$\text{logit}\left(\mu_{i,j}\right)=\alpha_{j}+\beta_{j}bare_{i,j}+\gamma_{j}bare_{i,j}^{2}+\delta_{j}height_{i,j}+\varepsilon_{j}height_{i,j}^{2}$$

The individual slope parameters are then modelled independently from each other with a normal distribution to estimate the population mean and variance of the slope parameters:

$$\alpha_{j}\sim N(\mu_{\alpha},\sigma_{\alpha}^{2})$$

$$\beta_{j}\sim N(\mu_{\beta},\sigma_{\beta}^{2})$$

$$\gamma_{j}\sim N(\mu_{\gamma},\sigma_{\gamma}^{2})$$

$$\delta_{j}\sim N(\mu_{\delta},\sigma_{\delta}^{2})$$

$$\varepsilon_{j}\sim N(\mu_{\varepsilon},\sigma_{\varepsilon}^{2})$$

The current model considered individual random effects for the intercept as well as individual random slopes, thus allows to model individual functional responses.

We specified non-informative priors for all parameters to be estimated. We used N(0, 1000) priors for the slope parameters and uniform priors U(0, 100) on the standard deviations for the variance parameters [1]. To calculate the posterior distributions of the parameters of interest, we used MCMC simulations implemented in WinBUGS [2] executed from R (R Development Core Team 2004) with package R2WinBUGS [3]. We ran 5 independent chains, with 50’000 iteration after 10’000 initial burn-in iterations, and checked the convergence using the Brooks–Rubin–Gelman diagnostic [4]. Convergence was satisfactory for all parameters (R < 1.05). We saved every 50^th^ MCMC sample and based inference on the remaining 1’000 posterior samples. For predictive graphs, we ran 1 chain with 110’000 iterations and a burn-in period of 10’000 iterations, and saved every 50^th^ observation. The explanatory variables were all standardized (mean = 0, sd = 1) prior to analysis.

**References**

1. Gelman A (2006) Prior distributions for variance parameters in hierarchical models. Bayesian Anal. 1: 515-534.
2. Lunn DJ, Thomas A, Best N, Spiegelhalter D (2000) WinBUGS – A Bayesian modelling framework: concepts, structure, and extensibility. Stat Comput 10: 325-337.
3. Sturtz S, Ligges U, Gelman A (2005) R2WinBUGS: a package for running WinBUGS from R. J Stat Softw 12: 1-16.
4. Brooks SP, Gelman A (1998) General methods for monitoring convergence of iterative simulations. J Comput Graph Stat 7: 434-455.
