# Supplementary figures and images for: Patches of Bare Ground as a Staple Commodity for Declining Ground-Foraging Insectivorous Farmland Birds
Source: PLoS One. 2010 Oct 6;5(10):e13115. doi: 10.1371/journal.pone.0013115 (PMC2950849; doi:10.1371/journal.pone.0013115)

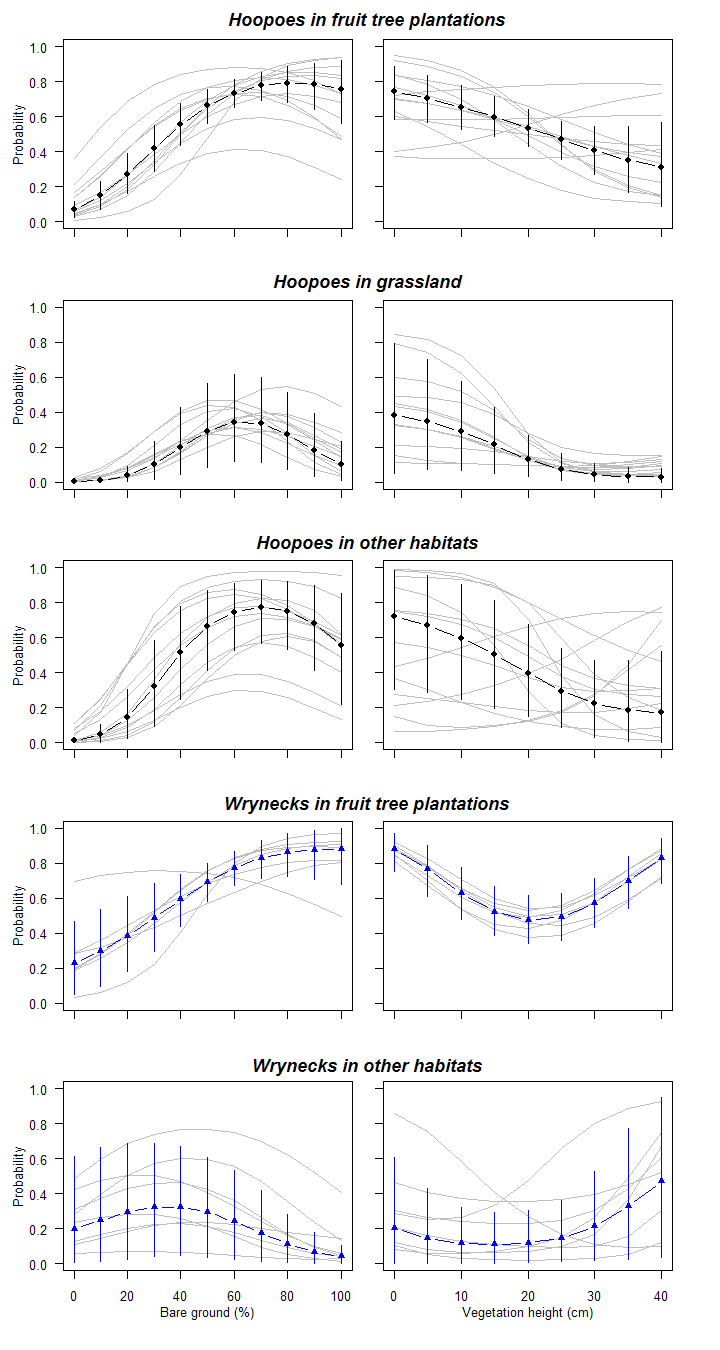

Supplement: Figure S1 — Selection probability of habitat use in relation to amount of bare ground and vegetation height for hoopoe and wryneck in different habitat categories as revealed by the most complex model. The grey lines show the individual effects, the black and blue line shows the population (marginal) average with 80% credible intervals. Note that selection probabilities below 0.5 indicate avoidance, selection probabilities above 0.5 indicate preference. (2.92 MB TIF) [file pone.0013115.s002.tif]

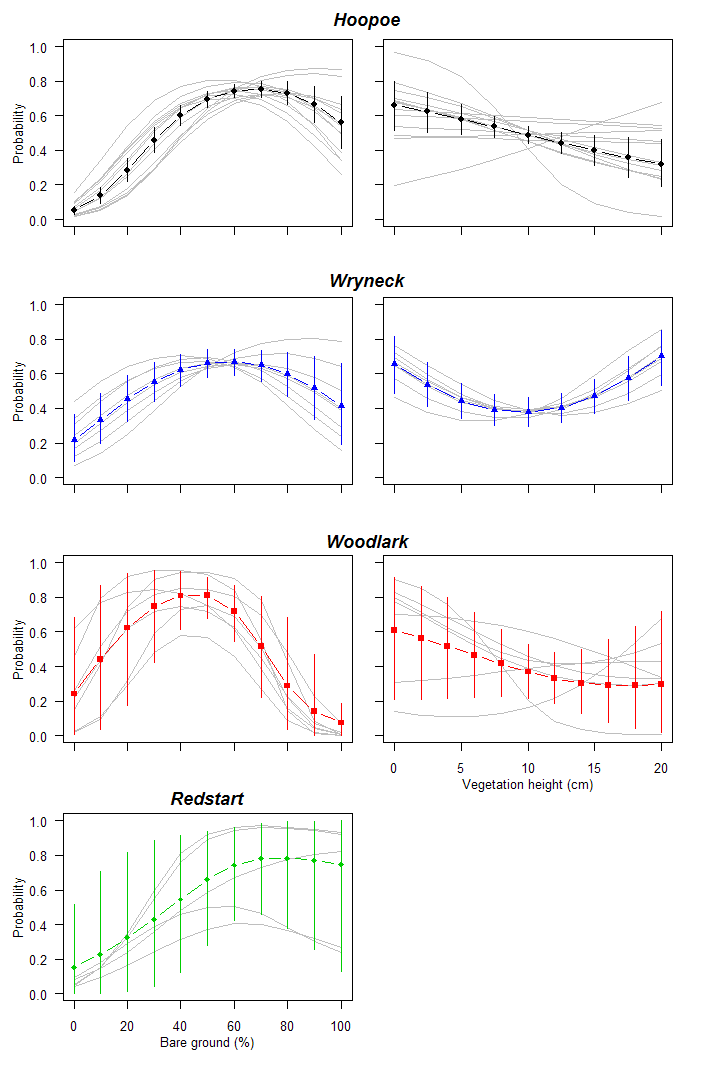

Supplement: Figure S2 — Selection probability of habitat use in relation to the amount of bare ground and vegetation height for four farmland species as revealed by the best models (Table 1). The grey lines show the individual effects, the coloured lines show the population (marginal) average with 80% credible intervals. Note that selection probabilities below 0.5 indicate avoidance, selection probabilities above 0.5 indicate preference. (2.33 MB TIF) [file pone.0013115.s003.tif]
