# Supplementary material for: Patches of Bare Ground as a Staple Commodity for Declining Ground-Foraging Insectivorous Farmland Birds
Source: PLoS One. 2010 Oct 6;5(10):e13115. doi: 10.1371/journal.pone.0013115 (PMC2950849; doi:10.1371/journal.pone.0013115)
Supplement: Table S1 — Sample sizes, locations and the use of radio-tags for the four studies: number of individuals, total number of observations and random points, and mean number of observations and random points per individual. (0.04 MB DOC) [file pone.0013115.s004.doc]

|  |  |  |  |  |
| --- | --- | --- | --- | --- |
|  | Hoopoe | Wryneck | Woodlark | Common redstart |
|  |  |  |  |  |
|  |  |  |  |  |
| Number of individuals (males) | 13 (13) | 8 (3, 2 unknown sex) | 7 (5) | 5 (3) |
| Total number of observation points (random points) | 552 (496) | 145 (141) | 504 (504) | 270 (270) |
| Mean number of observation points per individual (range) | 42 (10 – 55) | 18 (6 – 20) | 72 (58 – 96) | 54 (28 – 95) |
| Mean number of random points per individual (range) | 38 (9 – 54) | 18 (5 – 21) | 72 (58 – 96) | 54 (28 – 95) |
| Location | Valais | Valais | Valais | Basel |
| Radio tags used | Yes | Yes | Yes | No |
|  |  |  |  |  |
