# Supplementary material for: Patches of Bare Ground as a Staple Commodity for Declining Ground-Foraging Insectivorous Farmland Birds
Source: PLoS One. 2010 Oct 6;5(10):e13115. doi: 10.1371/journal.pone.0013115 (PMC2950849; doi:10.1371/journal.pone.0013115)
Supplement: Table S2 — Estimates of the mean model parameters and of their variability among individuals from the most complex model (b+b2+h+h2) for each species. Values in parentheses show the limits of the 80% credible intervals for each estimate. (0.05 MB DOC) [file pone.0013115.s005.doc]

|  |  |  |  |  |  |  |  |  |  |  |  |
| --- | --- | --- | --- | --- | --- | --- | --- | --- | --- | --- | --- |
|  | Hoopoe | |  | Wryneck | |  | Woodlark | |  | Common redstart | |
|  |  |  |  |  |  |  |  |  |  |  |  |
|  |  |  |  |  |  |  |  |  |  |  |  |
|  | Mean | Variance |  | Mean | Variance |  | Mean | Variance |  | Mean | Variance |
|  |  |  |  |  |  |  |  |  |  |  |  |
|  |  |  |  |  |  |  |  |  |  |  |  |
| Intercept | 0.933 (0.666; 1.216) | 0.498 (0.273; 0.762) |  | 0.290 (-0.107; 0.680) | 0.318 (0.039; 0.664) |  | 0.956 (0.442; 1.429) | 0.911 (0.453; 1.481) |  | 3.568 (1.824; 5.701) | 2.505 (0.342; 5.201) |
| Bare ground | 1.630 (1.345; 1.910) | 0.667 (0.430; 0.936) |  | 1.198 (0.664; 1.762) | 0.938 (0.421; 1.565) |  | -2.194 (-2.754; -1.658) | 1.050 (0.574; 1.640) |  | 3.032 (1.212; 5.213) | 3.054 (1.073; 5.622) |
| Bare ground ^2^ | -1.017 (-1.160; -0.874) | 0.144 (0.017; 0.318) |  | -0.856 (-1.238; -0.469) | 0.424 (0.066; 0.903) |  | -1.715 (-2.290; -1.183) | 1.128 (0.577; 1.744) |  | -3.322 (-5.979; -0.709) | 4.174 (1.213; 7.615) |
| Vegetation height | -0.499 (-0.912; -0.077) | 1.056 (0.663; 1.514) |  | 0.028 (-0.393; 0.448) | 0.718 (0.185; 1.332) |  | -0.688 (-2.183; 0.823) | 3.201 (1.331; 5.425) |  | - | - |
| Vegetation height^2^ | -0.026 (-0.188; 0.132) | 0.198 (0.037; 0.393) |  | 0.598 (0.209; 1.011) | 0.588 (0.105; 1.184) |  | -0.263 (-1.271; 0.509) | 1.542 (0.525; 2.843) |  | - | - |
|  |  |  |  |  |  |  |  |  |  |  |  |
